# Early prone positioning after tetralogy of fallot repair in children: a retrospective cohort study of oxygenation, safety, and short-term outcomes

**DOI:** 10.3389/fcvm.2026.1696761

**Published:** 2026-04-10

**Authors:** Xiaoqin Huang, Shaoping Su, Yang Du, Qi Liu, Zhanhao Su, Lixia Lin

**Affiliations:** 1 Department of the Intensive Care Unit of Cardiac Surgery, Guangdong Cardiovascular Institute, Guangdong Provincial People’s Hospital (Guangdong Academy of Medical Sciences), Southern Medical University, Guangzhou, China; 2 Guangdong Provincial Key Laboratory of South China Structural Heart Disease, Guangzhou, China; 3 Huizhou Central People’s Hospital, Huizhou, China; 4 Department of Organ Transplantation, Guangdong Provincial People’s Hospital (Guangdong Academy of Medical Sciences), Southern Medical University, Guangzhou, China; 5 Department of Cardiac Surgery, Guangdong Cardiovascular Institute, Guangdong Provincial People's Hospital, Southern Medical University, Guangzhou, China

**Keywords:** oxygenation index (PaO₂/FiO₂ ratio), pediatric cardiac surgery, prone positioning, tetralogy of fallot, ventilator-associated pneumonia

## Abstract

**Aim:**

To assess whether early prone positioning is associated with differences in gas-exchange indices at prespecified postoperative time points after complete tetralogy of Fallot (TOF) repair in children, without evidence of hemodynamic instability. We also explored associations between early respiratory indices and short-term outcomes.

**Methods:**

This was a single-center retrospective cohort study of patients aged <18 years undergoing complete TOF repair. Groups were defined by positioning during the first 12 postoperative hours: prone (*n* = 44) or supine (*n* = 43). PaO₂, PaCO₂, and PaO₂/FiO₂ were measured at 1, 4, 8, and 12 h; hemodynamics were recorded over 24 h; short-term outcomes within 60 days were ascertained, and postoperative mortality was assessed during in-hospital follow-up and at 3 months. Group comparisons, Pearson correlations, and receiver operating characteristic analyses were performed.

**Results:**

Prone positioning was associated with higher oxygenation indices vs. supine (higher PaO₂ at 1, 4, 8, 12 h; higher PaO₂/FiO₂ at 4, 8, 12 h) and with lower PaCO₂ at 8 and 12 h (all *P* ≤ 0.05). Hemodynamics were similar between groups, and no maneuver-related adverse events were documented. Short-term clinical outcomes did not differ. No in-hospital deaths occurred in either group, and no deaths were identified at the 3-month postoperative follow-up. Within the prone group, PaO₂/FiO₂ at 12 h correlated inversely with hospital stay (*r* = −0.33, *P* = 0.03). PaCO₂ at 8 h showed modest discrimination for ventilator-associated pneumonia (AUC 0.73; sensitivity 92.86%, specificity 31.25).

**Conclusions:**

Early short-duration prone positioning was associated with differences in gas-exchange indices at prespecified time points without evidence of hemodynamic instability after pediatric TOF repair, while short-term outcomes were similar. Findings support feasibility and warrant prospective multicenter evaluation to define optimal timing and duration.

## 1 Introduction

Tetralogy of Fallot (TOF) is the most common cyanotic congenital heart defect. Advances in diagnosis, perioperative care, and surgical techniques have produced excellent early and long-term outcomes in most patients. Despite these improvements, residual right ventricular outflow tract obstruction, pulmonary regurgitation, and arrhythmias remain common and often require reintervention (1–3). In addition, persistent or recurrent hypoxemia may occur after repair due to residual ventricular dysfunction, altered distribution of pulmonary and systemic blood flow, postoperative pulmonary edema, and changes in chest wall mechanics after sternotomy. These factors impair gas exchange and can delay liberation from mechanical ventilation (1–5).

Several perioperative factors have been linked to prolonged mechanical ventilation (PMV) after TOF repair. Reported predictors include small pulmonary artery dimensions (lower McGoon ratio or Nakata index), a history of palliative shunt, longer cardiopulmonary bypass and aortic cross-clamp times, a higher vasoactive-inotropic score (VIS), impaired left-ventricular diastolic function, younger age, lower body weight, and markers of chronic hypoxia (6–8). In a propensity-matched cohort, preoperative oxygen saturation below 90% was associated with higher rates of early extubation failure and PMV (7). Among children with chronic hypoxia, maintaining a lower postoperative left atrial pressure was associated with better pulmonary outcomes (7). Independently, a multicenter series identified lower preoperative pulse oximetry saturation as a predictor of PMV (9). These observations highlight the importance of optimizing early postoperative oxygenation following TOF repair in children.

Current evidence indicates a mortality benefit from early, prolonged prone positioning in adults with acute respiratory distress syndrome (ARDS) (10–13), likely through improved matching of ventilation and perfusion, recruitment of dependent lung, and a more homogeneous distribution of stress and strain (14, 15). Pediatric studies, although smaller, consistently show improved oxygenation under standardized protocols without a major increase in adverse events (16–19). However, sample size and methodological heterogeneity limit generalizability in children, which underscores the need for targeted assessment in high-risk postoperative populations such as those undergoing TOF repair. Accordingly, the feasibility and early physiological effects of prone positioning after TOF repair merit evaluation.

In this retrospective cohort of children undergoing complete TOF repair, we assessed whether early postoperative prone positioning was associated with gas-exchange indices at prespecified postoperative time points and hemodynamic tolerance. We also compared short-term outcomes and mortality between groups. We further explored the associations and predictive value of early respiratory indices (PaO₂/FiO₂ at prespecified time points and PaCO₂ trajectories) for predicting duration of mechanical ventilation, ICU length of stay, and ventilator-associated pneumonia.

## 2 Materials and methods

### 2.1 Study design and population

The relative benefit of prone vs. supine positioning in the early postoperative period remains uncertain. During the study period, prone positioning after complete TOF repair was available in the ICU as an adjunctive strategy for respiratory support but was not part of a formal standardized postoperative protocol or routine postoperative care. We conducted a single-center retrospective cohort study comparing outcomes in children undergoing complete TOF repair. Clinical data were extracted from the electronic medical records for consecutive patients treated at the Guangdong Cardiovascular Institute, Guangdong Provincial People's Hospital, Southern Medical University (Guangzhou, China), between October 7, 2018 and April 28, 2022. The study was approved by the Research Ethics Committee of Guangdong Provincial People's Hospital, Southern Medical University (GDREC2019338HR2) and conducted in accordance with the Declaration of Helsinki. Owing to the retrospective observational design, informed consent was waived.

Inclusion criteria were pediatric patients aged <18 years who underwent complete TOF repair, received invasive mechanical ventilation for at least the first 12 postoperative hours in the ICU, and had postoperative positioning documented. All children were initially admitted to the ICU in the supine position. Positioning during the first 12 postoperative hours was at the discretion of the attending intensivist, based on the child's overall respiratory and hemodynamic status. Prone positioning was considered as an adjunctive measure when oxygenation remained unsatisfactory despite ventilator adjustments and recruitment maneuvers, particularly in the presence of radiographic or clinical signs of atelectasis, and only in hemodynamically stable children without contraindications. In our center, prone positioning was performed by five trained and certified staff members. One physician at the head of the bed was responsible for airway protection and ventilator tubing. Two staff members were responsible for turning the patient and securing upper-body lines and drains. The remaining two staff members were responsible for lower-body line security and continuous monitoring throughout the maneuver. The turning procedure included moving the patient toward one side of the bed, organizing all lines and tubes with adequate slack, turning the patient around the bod*y* axis, and readjusting body position and devices after prone positioning was achieved. For analysis, patients were grouped according to their predominant position during this 12 h period: the prone group comprised children who were turned and maintained in the prone position for most of the first 12 h, whereas the supine group comprised those who remained supine throughout.

**Figure 1:**
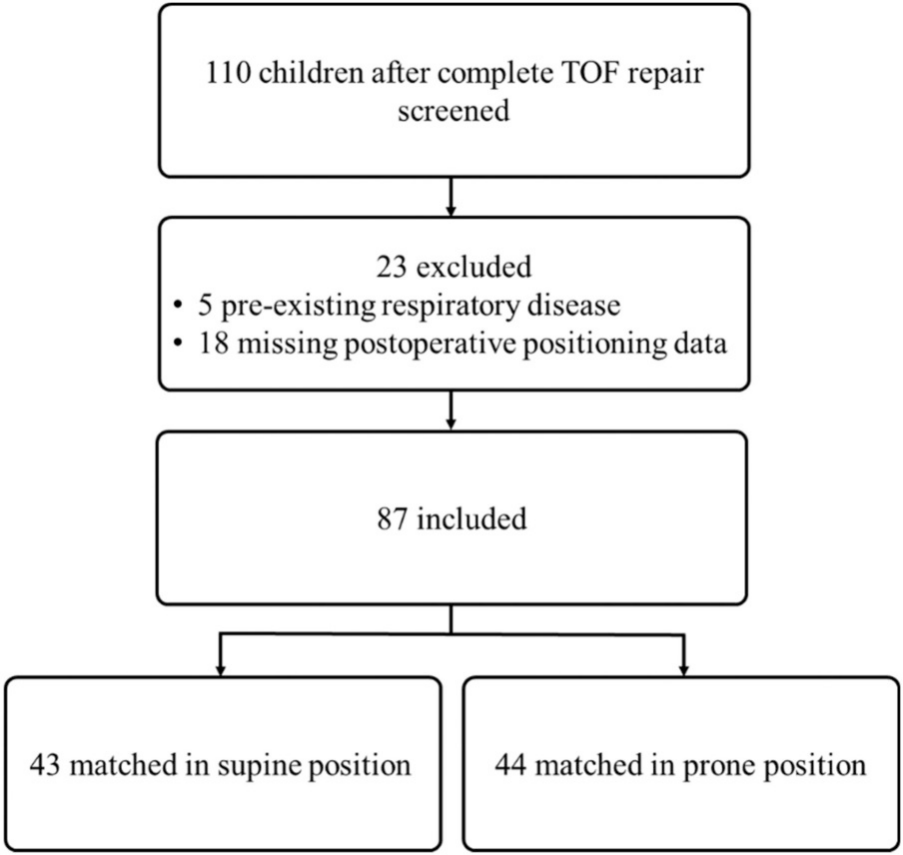
Study flow diagram of patient selection and group allocation. Of 110 children after complete TOF repair, 23 were excluded (5 with pre-existing respiratory disease; 18 with missing postoperative positioning data). The remaining 87 were categorized by positioning during the first 12 postoperative hours: supine (*n* = 43) and prone (*n* = 44).

Exclusion criteria were pre-existing respiratory disease at the time of surgery or ICU admission (including COVID-19 infection, bacterial or viral pneumonia, or respiratory failure) and missing postoperative positioning data. Of the 110 patients screened, 5 were excluded because of pre-existing respiratory disease and 18 owing to missing positioning data, leaving 87 patients for analysis (supine group, *n* = 43; prone group, *n* = 44; as shown in Figure 1).

### 2.2 Data collection

Baseline variables included age, sex, height, weight, body mass index (BMI), history of intensive care unit (ICU) admission, preoperative respiratory measures [pulse oximetry oxygen saturation (SpO₂), fraction of inspired oxygen (FiO₂), arterial partial pressures of oxygen and carbon dioxide (PaO₂, PaCO₂), and the PaO₂/FiO₂ ratio], the McGoon ratio, cardiopulmonary bypass time, aortic cross-clamp time, and preoperative hemoglobin. Preoperative PaO₂/FiO₂ ratios were calculated from arterial PaO₂ and the FiO₂ documented at the time of blood gas sampling and were not restricted to measurements on room air.

Early postoperative respiratory parameters were abstracted at 1, 4, 8, and 12 h within the first 12 postoperative hours in the ICU. At each time point, SpO₂, FiO₂, PaO₂, and PaCO₂ were recorded and the PaO₂/FiO₂ ratio was calculated. Postoperative hemodynamics recorded for the first 24 h included systolic and diastolic blood pressure (SBP, DBP), mean arterial pressure (MAP), heart rate (HR), central venous pressure (CVP), ejection fraction (EF), the VIS, and serum lactate. Short-term outcomes within 60 days after TOF repair included postoperative hospital length of stay (LOS, days), ICU LOS (days), duration of mechanical ventilation (hours), reintubation, pressure injury, ventilator-associated pneumonia (VAP), and use of extracorporeal membrane oxygenation (ECMO). In-hospital mortality and 3-month postoperative mortality were also recorded. Safety during prone positioning was assessed by reviewing nursing records, adverse-event reports, and medical records for maneuver-related complications, including accidental removal of lines, tubes, drains, or pacing wires, as well as kinking, compression, or drainage obstruction during turning. VAP was defined as a new or progressive pulmonary infiltrate on chest radiography together with at least two of the following: fever or hypothermia, leukocytosis or leukopenia, and increased purulent tracheal secretions, with no more likely alternative diagnosis. Respiratory cultures were obtained when possible to support, but not solely determine, the diagnosis. To explore prognostic relevance, we examined associations between early respiratory indices and clinical outcomes and performed receiver operating characteristic (ROC) analyses to assess the discriminative ability of PaO₂/FiO₂ at prespecified time points and PaCO₂ for these outcomes.

### 2.3 Statistical analysis

Normality was assessed with the Kolmogorov–Smirnov test. Continuous variables that were normally distributed are presented as mean ± standard deviation; those not normally distributed are presented as median and interquartile range. Categorical variables are presented as counts and percentages. Between-group comparisons were performed using the independent-samples t test, chi-square test, or Fisher's exact test, as appropriate. Variables that reached statistical significance were further evaluated with receiver operating characteristic (ROC) analysis. In exploratory analyses, PaCO₂ at 8 and 12 h was evaluated by ROC as a marker of subsequent VAP. Pearson correlation analysis was used to assess correlations. Statistical processing was performed with SPSS version 25.0 and MedCalc version 19.0. A two-sided *P* value < 0.05 was considered statistically significant.

## 3 Results

### 3.1 Clinical characteristics of pediatric patients undergoing TOF repair

Preoperative baseline characteristics of the supine and prone groups are summarized in Table 1. There were no statistically significant between-group differences in sex distribution, age, height, weight, body mass index, preoperative hemoglobin, prior ICU admission, preoperative oxygenation indices, arterial blood gases, McGoon ratio, cardiopulmonary bypass time, or aortic cross-clamping time (all *P* > 0.05). Preoperative hemoglobin was numerically higher in the supine group than in the prone group (median 138.0 vs. 124.5 g/L; *P* = 0.061), and age tended to be higher as well (8 vs. 6 months; *P* = 0.117), although neither difference reached statistical significance. Indices of gas exchange (SpO₂, PaO₂/FiO₂, PaO₂, PaCO₂) and pulmonary artery size (McGoon ratio) were closely matched between groups. Cardiopulmonary bypass and aortic cross-clamping times were comparable, suggesting similar operative complexity across groups.

**Table 1:** Preoperative clinical characteristics of the study cohort.

Variable	Supine (*n* = 43)	Prone (*n* = 44)	*P* value
Sex, male *n* (%)	27 (62.8)	31 (70.5)	0.596
Age, months	8.0 (6.0, 11.0)	6.0 (4.5, 12.0)	0.117
Height, cm	67.0 (64.0, 75.0)	68.0 (65.0, 73.0)	0.953
Weight, kg	7.5 (6.5, 8.5)	7.2 (6.1, 8.1)	0.548
Body mass index, kg/m²	15.6 (14.8, 16.6)	15.3 (13.6, 16.9)	0.262
Preoperative hemoglobin, g/L	138.0 (120.5, 152.5)	124.5 (110.0, 147.5)	0.061
Prior ICU admission, *n* (%)	2 (4.7)	3 (6.8)	0.664
Preoperative SpO₂, %	85.0 (75.5, 95.0)	83.5 (72.0, 92.5)	0.415
Preoperative PaO₂/FiO₂ ratio	188.0 (155.0, 220.0)	182.5 (162.0, 211.5)	0.757
McGoon ratio	1.6 (1.4, 1.7)	1.5 (1.4, 1.8)	0.935
Preoperative PaCO₂, mmHg	45.0 (33.5, 48.0)	41.5 (34.0, 46.5)	0.546
Preoperative PaO₂, mmHg	89.0 (76.0, 98.0)	84.0 (78.0, 94.5)	0.475
Cardiopulmonary bypass time, min	125.0 (104.5, 179.5)	126.0 (114.5, 163.0)	0.832
Aortic cross-clamp time, min	77.0 (62.5, 96.0)	75.0 (60.5, 94.0)	0.498

Preoperative PaO₂/FiO₂ ratios were calculated from arterial PaO₂ and the concurrent FiO₂ and were not restricted to room air measurements.

### 3.2 Association of prone positioning with postoperative oxygenation and carbon dioxide retention after TOF repair

**Figure 2:**
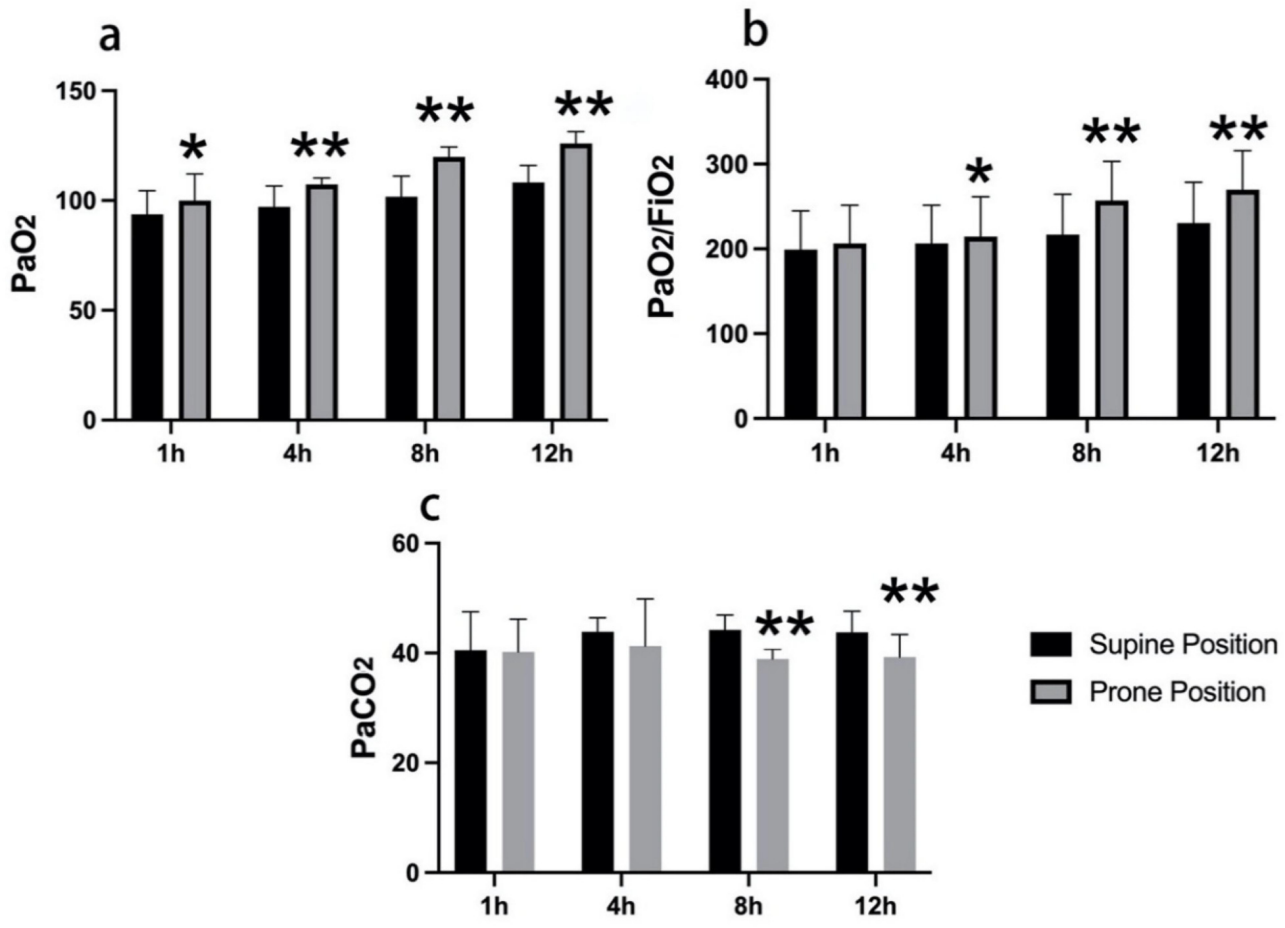
Early postoperative respiratory indices by positioning after TOF repair. **(a)** Arterial oxygen tension (PaO₂). **(b)** PaO₂/FiO₂ ratio. **(c)** Arterial carbon dioxide tension (PaCO₂). Measurements were obtained at 1, 4, 8, and 12 h after ICU admission. Black bars indicate the supine group; gray bars indicate the prone group. Error bars indicate standard deviation. *P < 0.05; **P < 0.01 versus supine at the same time point.

Respiratory indices were compared between the prone and supine groups at 1, 4, 8, and 12 h in the ICU. Prone positioning was associated with higher oxygenation indices and lower PaCO₂ relative to supine positioning. PaO₂ was higher in the prone group at 1 h (*P* < 0.05) and at 4, 8, and 12 h (all *P* < 0.01) (Figure 2a). The PaO₂/FiO₂ ratio was also higher in the prone group at 4 h (*P* < 0.05) and at 8 and 12 h (both *P* < 0.01) (Figure 2b). PaCO₂ was lower in the prone group at 8 and 12 h (both *P* < 0.01) (Figure 2c).

Across the first 12 postoperative hours, both groups showed gradual changes in oxygenation. Between-group differences in PaO₂ were present from 1 h onward, whereas differences in the PaO₂/FiO₂ ratio emerged at 4 h and persisted through 8 and 12 h. PaCO₂ decreased over time in both groups, with the largest between-group differences observed at 8 and 12 h. Variability appeared comparable between groups at each time point.

### 3.3 Postoperative hemodynamics by positioning

**Figure 3:**
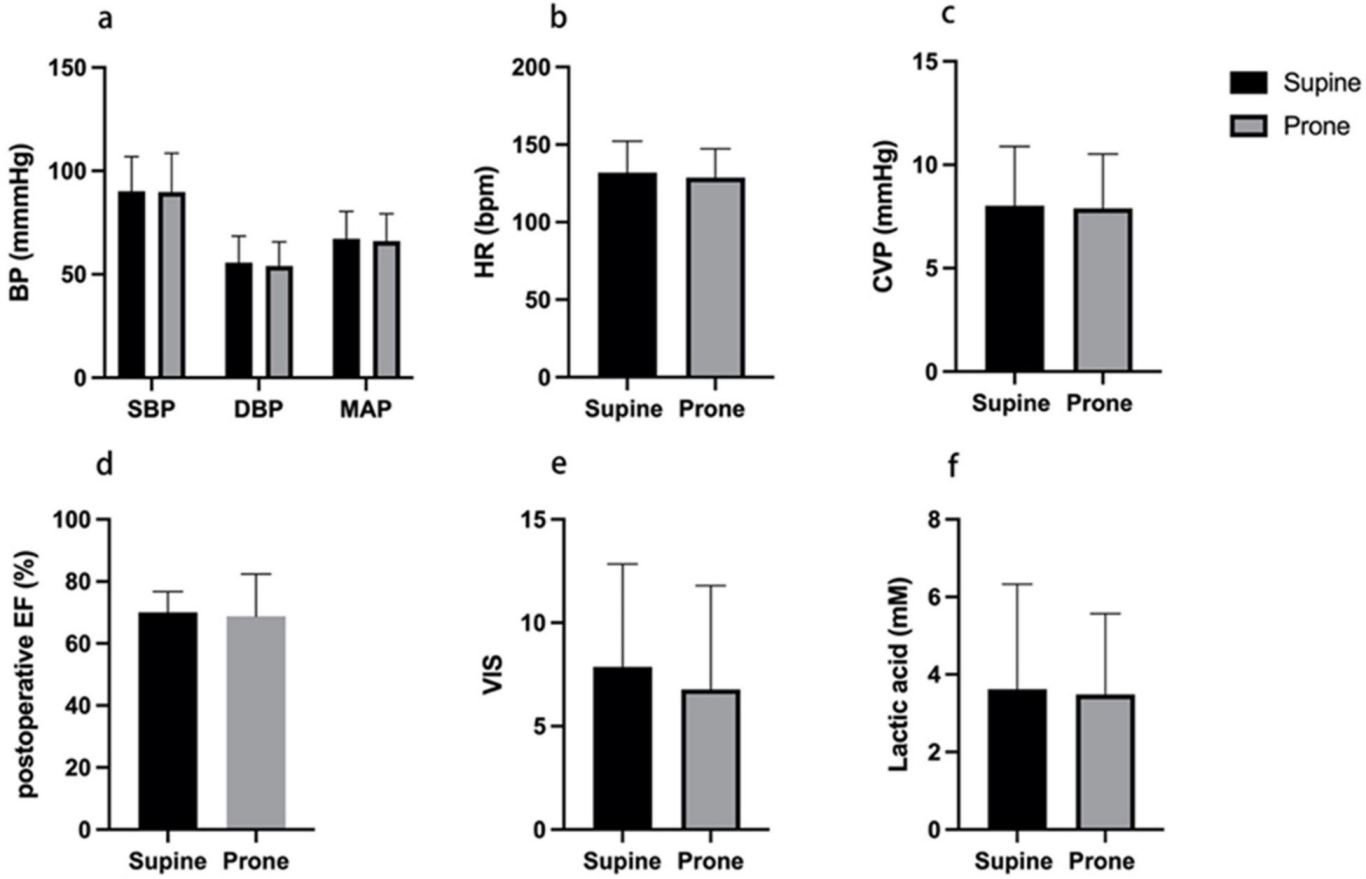
Early postoperative hemodynamics by positioning after TOF repair. Panels depict: **(a)** systolic, diastolic, and mean arterial pressure; **(b)** heart rate; **(c)** central venous pressure; **(d)** postoperative ejection fraction; **(e)** VIS; and **(f)** serum lactate. Measurements reflect the first 24 postoperative hours in the ICU. Black bars indicate the supine group and gray bars indicate the prone group. Error bars denote variability. No significant between-group differences were observed.

Early postoperative hemodynamics were compared between the prone and supine groups. Systolic and diastolic blood pressure, mean arterial pressure, heart rate, central venous pressure, ejection fraction, VIS, and serum lactate in the first 24 h did not differ significantly between groups (all *P* > 0.05). Across the first 24 postoperative hours, blood pressure profiles were comparable with overlapping variability for SBP, DBP, and MAP (Figure 3a). Heart rate and central venous pressure were likewise similar (Figures 3b,c). Left ventricular systolic function remained preserved in both groups with comparable postoperative ejection fraction (Figure 3d). Requirements for vasoactive support did not differ. The prone group showed numerically lower VIS without statistical significance (Figure 3e). Serum lactate was also numerically lower in the prone group, again without statistical significance (Figure 3f). Overall, no between-group differences reached statistical significance.

### 3.4 Clinical outcomes by positioning after TOF repair

We compared short-term outcomes between the prone and supine groups, as summarized in Table 2. Outcomes included hospital LOS, ICU LOS, duration of invasive mechanical ventilation, reintubation, pressure injury, VAP, use of ECMO, in-hospital mortality, and 3-month postoperative mortality. Descriptively, hospital LOS was similar, with a median of 21.5 days in the prone group vs. 19.0 days in the supine group. ICU LOS had the same median in both groups (4.0 days), with a narrower interquartile range in the prone group (2.0–6.5 days, compared with 3.0–11.5 days in the supine group). The duration of invasive mechanical ventilation was numerically lower in the prone group (36.0 h vs. 58.0 h). Reintubation and VAP were also numerically lower in the prone group (6.8% vs. 9.3% and 36.4% vs. 44.2%, respectively), whereas pressure injury and ECMO use were numerically higher (13.6% vs. 9.3% and 4.5% vs. 2.3%). No in-hospital deaths occurred in either group, and no deaths were identified at the 3-month postoperative follow-up. In addition, no maneuver-related adverse events were documented during turning.

**Table 2:** Clinical outcomes after TOF repair.

Variable	Supine (*n* = 43)	Prone (*n* = 44)	*P* value
Hospital LOS, days	19.0 (15.0, 25.0)	21.5 (15.0, 30.0)	0.452
ICU LOS, days	4.0 (3.0, 11.5)	4.0 (2.0, 6.5)	0.144
Mechanical ventilation, hours	58.0 (21.5, 142.5)	36.0 (21.5, 129.0)	0.616
VAP, *n* (%)	19 (44.2)	16 (36.4)	0.457
ECMO, *n* (%)	1 (2.3)	2 (4.5)	0.570
Reintubation, *n* (%)	4 (9.3)	3 (6.8)	0.670
Pressure injury, *n* (%)	4 (9.3)	6 (13.6)	0.526
In-hospital mortality, *n* (%)	0 (0.0)	0 (0.0)	—
3-month postoperative mortality, *n* (%)	0 (0.0)	0 (0.0)	—

### 3.5 Association between hospital LOS and the PaO₂/FiO₂ ratio at 8 and 12 h in the prone group

**Figure 4:**
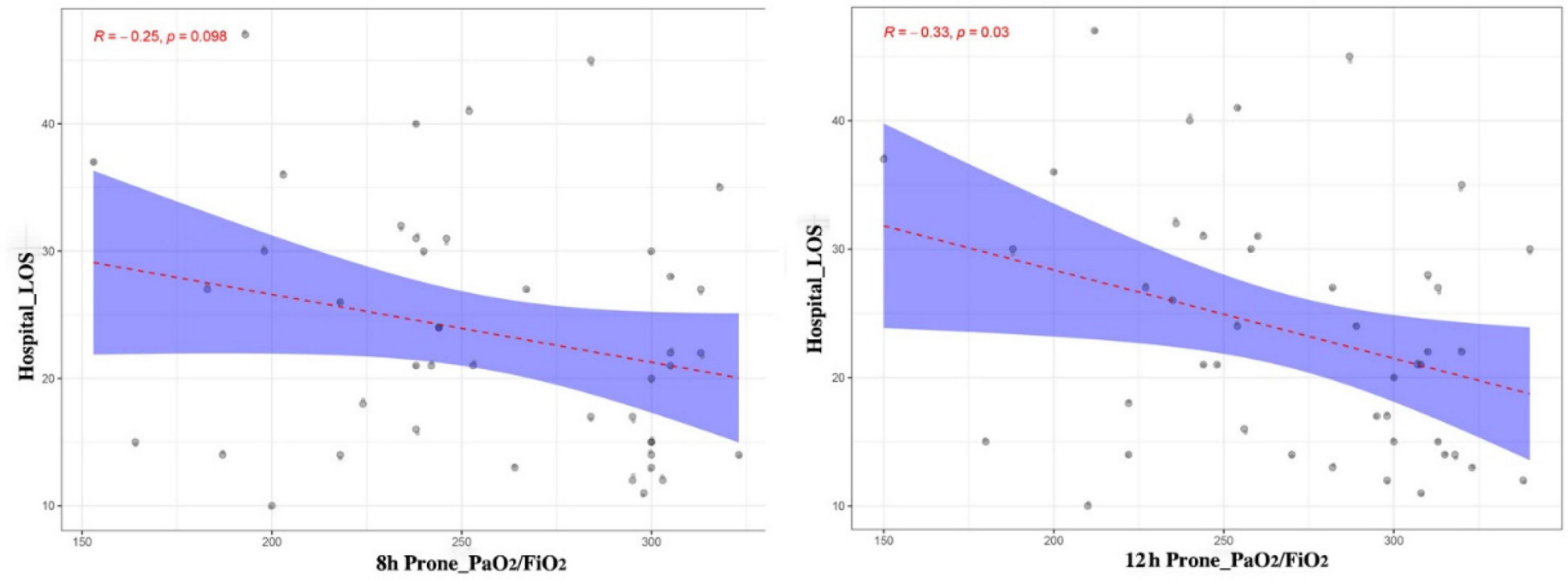
Association between hospital LOS and the PaO₂/FiO₂ ratio in the prone group after TOF repair. **(a)** Scatter plot of hospital length of stay versus PaO₂/FiO₂ at 8 h with fitted regression line; Pearson correlation *r* = −0.25, *P* = 0.098. **(b)** Scatter plot of hospital length of stay versus PaO₂/FiO₂ at 12 h; *r* = −0.33, *P* = 0.03. Shading indicates the confidence band around the fitted line.

To examine the relationship between early respiratory indices and clinical outcomes, we assessed Pearson correlations within the prone group. At 8 h, hospital length of stay showed a negative correlation with the PaO₂/FiO₂ ratio that did not reach statistical significance (*r* = −0.25, *P* = 0.098; Figure 4a), indicating a weak inverse association. At 12 h, the negative association strengthened and achieved statistical significance (*r* = −0.33, *P* = 0.03; Figure 4b). These findings suggest that higher PaO₂/FiO₂ values at 12 h were associated with shorter hospital stay in the prone group.

### 3.6 ROC analysis of postoperative PaCO₂ for predicting VAP

**Figure 5:**
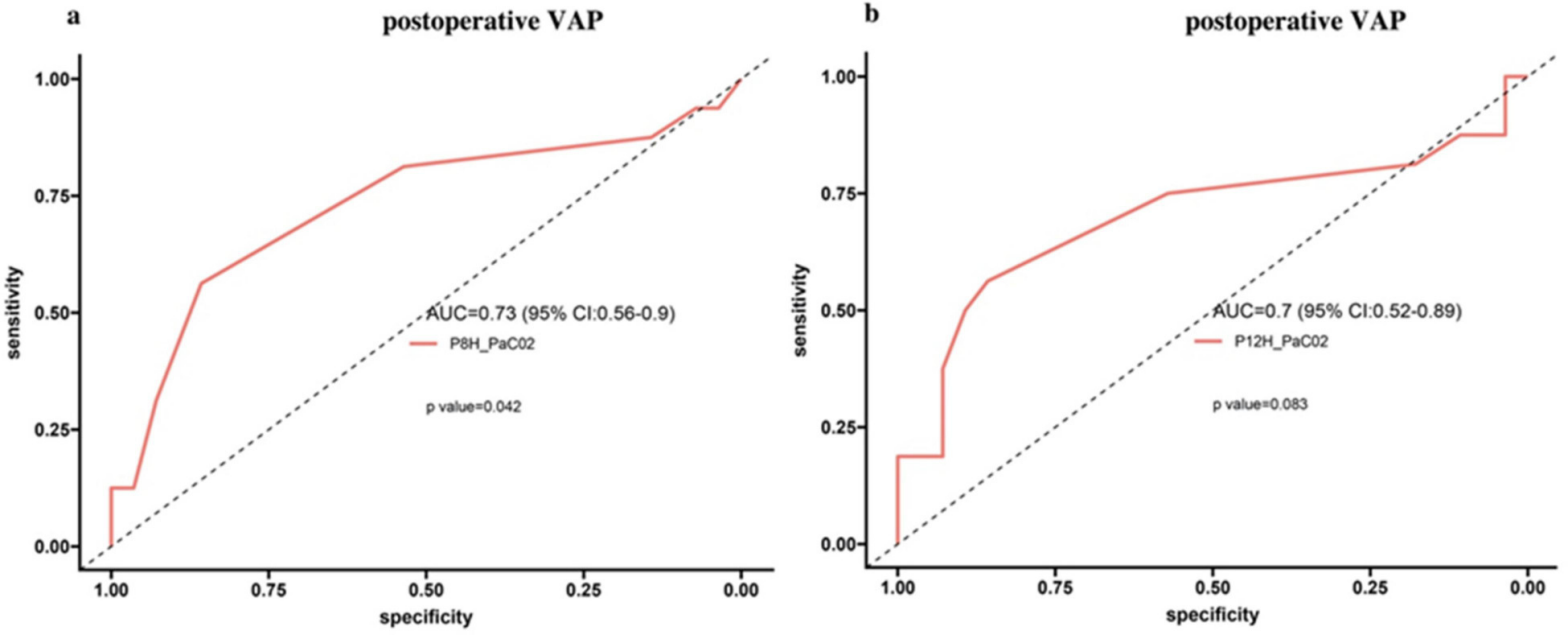
Receiver operating characteristic analysis of PaCO₂ during prone positioning for predicting postoperative VAP after TOF repair. **(a)** PaCO₂ at 8 h: AUC 0.73 (95% CI, 0.56 to 0.90), *P* = 0.042; sensitivity 92.86%, specificity 31.25%. **(b)** PaCO₂ at 12 h: AUC 0.70 (95% CI, 0.52 to 0.89), *P* = 0.083; sensitivity 92.86%, specificity 37.5%. The dashed line indicates the line of no discrimination.

We evaluated whether early PaCO₂ measured during prone positioning could discriminate subsequent VAP after TOF repair, as shown in Figure 5. The ROC curve for PaCO₂ at 8 h lay above the line of no discrimination and showed modest performance with an AUC of 0.73 (95% CI, 0.56–0.90; *P* = 0.042). At the selected cutoff, sensitivity was 92.86% and specificity was 31.25% (Figure 5a). For 12 h, the ROC curve also trended above the diagonal with an AUC of 0.70 (95% CI, 0.52–0.89; *P* = 0.083); sensitivity was 92.86% and specificity 37.5% (Figure 5b). Overall, PaCO₂ at 8 h showed modest discriminative ability for VAP, whereas the result at 12 h did not reach statistical significance. These findings should be interpreted as exploratory.

## 4 Discussion

Early postoperative hypoxemia after TOF repair is common, yet pediatric evidence specific to prone positioning in this context is limited and practice varies across centers. We examined whether brief early prone positioning was associated with differences in gas-exchange indices between groups at prespecified postoperative time points, without evidence of hemodynamic instability. We also explored whether early respiratory indices were associated with short-term outcomes. During the study period, supine positioning was routine postoperative care, whereas prone positioning was used as an adjunctive strategy at the discretion of the attending intensivist and bedside team, based on the child's overall respiratory status and clinical condition, and was not mandated by a standardized protocol.

In our cohort, prone positioning was associated with higher oxygenation indices during the first 12 postoperative hours, with higher PaO₂ at 1, 4, 8, and 12 h, higher PaO₂/FiO₂ at 4, 8, and 12 h, and lower PaCO₂ at 8 and 12 h (Figure 2). Hemodynamics, vasoactive requirements, and lactate remained similar between groups (Figure 3). These time-course patterns align with contemporary physiologic studies that demonstrate improved dorsal ventilation, better ventilation–perfusion matching, and reduced shunt during proning, including bedside electrical impedance tomography work in ventilated adults and in awake, non-intubated patients (20, 21). The fall in PaCO₂ is consistent with improved alveolar ventilation and reduced physiologic dead space, a plausible effect after sternotomy where dependent atelectasis and altered chest wall mechanics can impede ventilation (20).

Safety signals were reassuring. We observed no excess in blood pressure instability, central venous pressure change, ejection fraction reduction, VIS, lactate, reintubation, or pressure injury in the prone group (Figure 3 and Table 2). In addition, no maneuver-related adverse events were documented during turning, and no in-hospital deaths or deaths at the 3-month postoperative follow-up were identified in either group. These findings are consistent with a recent cohort of postoperative cardiac surgery adults with ARDS in which early proning was associated with higher oxygenation, shorter ventilation and ICU stay, and no procedure-related complications, even among patients with intra-aortic balloon pump support (22).

Short-term clinical outcomes in our study, including hospital and ICU length of stay, duration of mechanical ventilation, ventilator-associated pneumonia, and extracorporeal membrane oxygenation use, did not differ significantly between groups (Table 2). These analyses were unadjusted and may be influenced by residual confounding, so the findings for short-term outcomes should be interpreted as exploratory. Thus, although prone positioning was associated with improved gas exchange, this did not correspond to measurable differences in short-term clinical outcomes in our cohort. This lack of separation is consistent with findings from adult ARDS cohorts, where longer daily proning sessions are associated with better outcomes. Similar results have also been reported in randomized studies of awake hypoxemic adults, in which sufficiently prolonged, protocolized exposure reduced the need for intubation (23). Our exposure was brief and restricted to the first postoperative 12 h, so any physiologic advantage may be diluted by multifactorial postoperative determinants of length of stay and complications. Early postoperative hemodynamics after TOF repair are also influenced by multiple factors, including myocardial recovery, loading conditions, vasoactive support, pulmonary vascular status, and ventilatory management. Low event counts for some endpoints also limit power to detect modest effects. Arterial blood gases were obtained at fixed postoperative time points rather than systematically immediately before each position change. Consequently, an immediate pre-/post-positioning comparison was not available. Immediate within-patient changes in gas exchange at the time of the intervention could not be assessed. This was a single-center study with a relatively small sample size (44 patients in the prone group and 43 in the supine group). Positioning was determined by clinical judgment rather than randomization. As a result, selection bias and confounding by indication are possible. The findings are exploratory and need to be confirmed in larger multicenter studies. Follow-up was limited to 3 months, and longer-term events such as late reinterventions, functional status, and quality of life could not be assessed.

Within the prone group, higher PaO₂/FiO₂ at 12 h correlated with shorter hospital stay, whereas no such association was observed at 8 h (Figure 4). Receiver operating characteristic analysis indicated that PaCO₂ at 8 h provided high sensitivity but limited specificity for ventilator-associated pneumonia (Figure 5). These findings should be regarded as exploratory. PaCO₂ may be useful as an adjunctive indicator rather than a stand-alone predictor, in line with physiologic studies that recommend integrating gas exchange metrics with clinical examination and microbiology before management decisions (20, 21).

Taken together, our data suggest that early prone positioning is a feasible adjunct for children with suboptimal oxygenation after TOF repair. Its implementation should be supported by a standardized bundle that includes careful padding, securement of endotracheal tubes and invasive lines, and coordinated team training. However, the present findings support feasibility and early physiological benefit rather than a demonstrable improvement in short-term clinical outcomes. Given the exposure–response signals in adult ARDS and the reassuring safety findings reported after cardiac surgery, future pediatric work should test protocolized, longer or repeated prone sessions, incorporate physiologic monitoring where feasible, and evaluate patient-centered outcomes in adequately powered, multicenter designs.

## 5 Conclusion

In this single-center retrospective cohort of children after TOF repair, early short-duration prone positioning was feasible without evidence of hemodynamic instability. Compared with supine positioning, the prone group showed differences in gas-exchange indices at prespecified postoperative time points over the first 12 postoperative hours, while short-term clinical outcomes were similar between groups. Within the prone cohort, higher PaO₂/FiO₂ at 12 h was associated with shorter hospital stay, and PaCO₂ at 8 h showed high sensitivity but limited specificity for ventilator-associated pneumonia, supporting its exploratory value as an adjunctive indicator rather than a stand-alone predictor. These findings support the feasibility of early prone positioning for children with suboptimal oxygenation after TOF repair. Prospective multicenter studies with adequate sample size and longer follow-up would be valuable to confirm these associations and guide protocol development for prone positioning after pediatric TOF repair.

## Data Availability

The raw data supporting the conclusions of this article will be made available by the authors, without undue reservation.
